# The facilitators and barriers to implementing patient reported outcome measures in organisations delivering health related services: a systematic review of reviews

**DOI:** 10.1186/s41687-018-0072-3

**Published:** 2018-10-03

**Authors:** Alexis Foster, Liz Croot, John Brazier, Janet Harris, Alicia O’Cathain

**Affiliations:** School of Health and Related Research, University of Sheffield, Regents Court, Regents Street, S1 4DA, Sheffield, UK

**Keywords:** Patient-reported outcomes, Quality of life, Outcome assessment, Implementing

## Abstract

**Background:**

There is increasing interest in using Patient Reported Outcome Measures (PROMs) within organisations delivering health related services. However, organisations have had mixed success in implementing PROMs and there is little understanding about why this may be. Thus, the purpose of this study was to identify the facilitators and barriers to implementing PROMs in organisations.

**Method:**

A systematic review of reviews was undertaken. Searches were conducted of five electronic databases: MEDLINE, EMBASE, CINAHL, PsycINFO and the Cochrane Database of Systematic Reviews, during the week of the 20th February 2017. Additional search methods included website searching and reference checking. To be included, a publication had to be a review of the literature, describe its methods and include information related to implementing PROMs. The reviews were extracted using a standardised form and assessed for their risk of bias using the Risk of Bias in Systematic Reviews tool. The findings were synthesised using the Consolidated Framework for Implementation Research. The protocol was registered on the International Prospective Register of Systematic Reviews database (PROSPERO) (CRD42017057491).

**Results:**

Initially 2047 records were identified. After assessing eligibility, six reviews were included. These reviews varied in their review type and focus. Different issues arose at distinct stages of the implementation process. Organisations needed to invest time and resources in two key stages early in the implementation process: ‘designing’ the processes for using PROMs within an organisation; and ‘preparing’ an organisation and its staff. The ‘designing’ stage involved organisations planning not just which PROMs to use and how to administer them, but also how the data would be used for clinical purposes. The ‘preparing’ stage involved getting an organisation and its staff ready to use PROMs, particularly persuading clinicians of the validity and value of PROMs, delivering training, and developing electronic systems. Having an implementation lead overseeing the process and developing the process based on feedback were also identified as facilitating implementation.

**Conclusion:**

Organisations implementing PROMs need to invest time and resources in ‘designing’ the PROMs strategy and ‘preparing’ the organisation to use PROMs. Focusing on these earlier stages may prevent problems arising when PROMs are used in practice.

**Electronic supplementary material:**

The online version of this article (10.1186/s41687-018-0072-3) contains supplementary material, which is available to authorized users.

## Background

Patient Reported Outcome Measures (PROMs), such as health-related quality of life measures, are questionnaires which measure Patient Reported Outcomes (PROs), such as a person’s perspective on their health, wellbeing or symptoms [1–3]. There is increasing interest in using PROMs routinely within healthcare organisations to evaluate clinical practice, audit clinical performance and/or to support the care management of individual patients [4]. For example, the national PROMs programme in the United Kingdom (UK) mandates that all hospitals utilise PROs for specific healthcare interventions [1]. In the United States of America (USA), the Patient Reported Outcomes Measurement Information System (PROMIS) programme is implementing PROs in clinical practice [5].

A key driver for using PROMs is to improve patient satisfaction and clinical outcomes by improving communication and shared decision-making between patients and clinicians [6–8]. Despite this motivation, organisations have had mixed experiences implementing PROMs. Implementation encompasses the tasks that are undertaken between an organisation deciding to use PROMs and PROMs becoming part of routine practice. Tasks include choosing which PROM to use, training clinicians and developing reporting systems.

Different frameworks can be used to operationalise the concept of implementation. In this review the Consolidated Framework for Implementation Research (CFIR) was selected because it distils the key constructs from a number of implementation theories [9], and it is not context specific so can be used in different settings [10]. The CFIR consists of five domains, each with a number of constructs which focus on different aspects of the domain and encapsulate different issues throughout the implementation pathway [10] (detailed in Table 1). Implementation research often organises issues within a barriers and facilitators framework [11] showing what constrains or enables an intervention to be implemented in an organisation. Thus, the CFIR provides a useful basis for classifying barriers and facilitators to implementation [11, 12]. Reported facilitators and barriers may be the result of how stakeholders have made sense of a situation, rather than these factors being the cause of successful or unsuccessful implementation [13]. For example, clinicians may perceive a barrier to be a lack of training but receiving further training may not necessarily improve the implementation of PROMs. Nonetheless, understanding of reported facilitators and barriers to implementation of PROMs may help organisations implement PROMS.

**Table 1:** Domains of the CFIR

Domain	Description	Example constructs
The intervention	In this case the design of the PROMs and associated processes for administering, analysing and using the data collected.	• Intervention source • Adaptability • Design
Outer setting	Factors external to the organisation which may impact on implementation. This includes the needs of patients that access the organisation.	• Patients’ needs and resources • Peer pressure • External policy and incentives
Inner setting	Factors internal to the organisation which may impact on implementation. For example available resources.	• Structural characteristics • Culture • Implementation climate
Characteristics of individuals	The impact of the views and behaviours of individuals within the organisation on implementation.	• Knowledge and beliefs about the intervention • Individuals stage of change • Individual identification with an organisation
Process	In this case, issues related to implementing PROMs such as evaluating the success of implementation.	• Planning • Engaging • Executing

Barriers to implementing PROMs include clinicians believing they do not have the capacity to use them [14, 15], clinicians perceiving their practice is being judged on changes in PROMs scores [16], organisations not having the resources to utilise PROMs, such as no administrative support [17] and organisations not incorporating PROMs into existing workflows [18]. There are also factors which facilitate implementation. Examples include choosing PROMs that clinicians feel are relevant to their patients [19], clinicians receiving feedback on their patients’ scores [20] and organisations providing sufficient training and support to staff on using PROMs [21].

Having a greater understanding of the issues which may impact on implementation will be useful for stakeholders wanting to use PROMs. To date, there have been a number of reviews on the implementation of PROMs [22, 23]. However they all focus on a particular area of healthcare, such as palliative care [22], or on a specific stage of the PROMs process, for example the feedback of scores [23]. Consequently, there is a need to synthesise these reviews to understand the issues across different contexts and at different stages of the PROMs process. Lessons learnt in specific organisations may be applicable elsewhere as the boundaries of healthcare provision are expanding [24]. For example, increasingly charities and social care providers are delivering health related services and are interested in using PROMs within their own service delivery [25]. Therefore, the aim of this study was to conduct a systematic review of reviews to identify the facilitators and barriers to implementing PROMs in organisations delivering health related services.

## Method

Throughout the review, the Preferred Reporting Items for Systematic Reviews and Meta-Analyses (PRISMA) guidance was followed]. The completed PRISMA checklist is included in Additional file 1. The protocol was registered on the International Prospective Register of Systematic Reviews database (PROSPERO) (CRD42017057491).

### Review methodology: A systematic review of reviews

Systematic reviews of reviews involve the same processes as systematic reviews of primary research including searching, sifting, data extraction, quality appraisal and synthesis [26]. However, the unit of analysis is a review rather than an individual study.

### Eligibility criteria

The search sought to identify published reviews of the literature which consider factors that impact on the implementation of PROMs in organisations delivering health related services. The search did not focus specifically on reviews of facilitators and barriers because these terms are not always used by researchers when reporting studies.

The following criteria were developed to frame the review:

- **Population-** Patients, clinicians, commissioners or managers of health-related services. Commissioners are representatives of local and national agencies that fund or finance health-related services, for example policy-makers.
- **Interest:** Issues reported as impacting on the implementation of PROMs.
- **Context:** Health related services irrespective of the type of provider or country.
- **Study type(s):** Reviews that provide a description of the methods used to conduct the review. They may classify themselves as a specific type of review e.g. a systematic review, narrative review, meta-analysis, meta-synthesis or scoping review.

To be included in the review, a publication had to meet all of the following inclusion criteria:

1. Be a review of the literature and provide a description of its methods [27].
2. Include information related to implementing PROMs.
3. Focus on health-related services irrespective of the type of provider.
4. Be published before February 2017.

Publications were excluded if they were:

1. Written in a language other than English. This was due to resource constraints.
2. Focused on the measurement properties of PROMs.
3. Focused on the results of PROMs e.g. when evaluating interventions.
4. Not focused on factors that impacted on the implementation of PROMs.

These latter criteria were to ensure that any included reviews focused on the implementation of PROMs.

### Search strategy

A comprehensive search strategy was developed by the review team in conjunction with an information specialist and performed by the primary reviewer (AF). Searches were conducted in five electronic databases: MEDLINE, EMBASE, CINAHL, PsycINFO and the Cochrane Database of Systematic Reviews during the week of the 20th February 2017. The search strategy for MEDLINE is detailed in Additional file 2. All databases were searched from inception. Some of the search terms related to settings such as social care and charities, in recognition of the diversity of providers delivering health related services [24]. The reference lists of the included reviews were screened for additional literature. To identify grey literature, the websites of UK based relevant organisations were searched including PROSPERO, the Kings Fund, NHS England, Social Care Institute for Excellence, the University of Birmingham Centre for Patient Reported Outcome Research and the National Council for Voluntary Organisations. Five researchers who were topic specialists and known to the authors were asked about relevant reviews. The grey literature search was UK based because the review was part of a wider study based on the UK context.

### Study selection

Studies were selected following established guidance [28]. Firstly, duplicate references were deleted. Secondly, AF screened all citations (titles and abstracts) for inclusion. A second reviewer (LC) independently screened 20% of the citations. AF and LC discussed their results and found they were highly consistent (inter-rater reliability of 95.6%). Therefore, full double screening of all the citations was deemed unnecessary. Thirdly, two reviewers (AF and LC) assessed the full text of potentially eligible reviews. They compared their results and had an inter-rater reliability of 86.2%. AF and LC discussed the reviews they disagreed on with other co-authors and consensus was reached on which reviews to include.

### Data extraction

A data extraction form was developed. It was tested on two of the included reviews and refined, particularly in relation to collecting greater information on the individual studies included in each review. The finalised form included the following categories:

- Title
- Aims/Objectives
- Checklist against the inclusion and exclusion criteria
- Checklist against the ROBIS
- Focus of the review
- Context
- Population
- Review type
- Review methodology e.g. type of synthesis method
- Number of included studies
- Lead author, year of publication, study type and focus of each of the included studies
- Issues affecting the implementation of PROMs.

To address differences in terminology amongst the reviews, the review team extracted any factors described by the authors of included reviews as impacting on the implementation of PROMs, rather than only those specifically labelled as facilitators or barriers.

AF conducted extraction for all the included reviews. LC conducted extraction on half of the included reviews. AF and LC compared their results to ensure consistency.

### Risk of bias

The Risk of Bias in Systematic Reviews tool (ROBIS) was utilised because it can be used to appraise a review, irrespective of the type of primary studies included [29]. As the reviews included a range of study designs, other tools such as the AMSTAR [30], which is designed to appraise reviews of Randomised Controlled Trials, were not appropriate. The ROBIS enables the user to consider potential issues in a review in terms of the eligibility criteria, the identification and selection of studies, data extraction, quality appraisal and synthesis]. AF assessed all included reviews and LC assessed half of these. As results were similar, no further double assessment was conducted. Reviews were not excluded based on the outcome of the ROBIS.

### Synthesis of results

Information extracted on the context and objectives of the reviews was used to contextualise them. Framework synthesis using the CFIR was used to make sense of the data extracted on issues impacting on implementation. The process of framework synthesis initially involved familiarisation with the data by reading the data extraction multiple times. Secondly, AF categorised the extracted data into the different constructs of the CFIR which produced a summary of the issues impacting on the implementation of PROMs [31, 32]. Whether an issue was coded as a facilitator or a barrier was determined by AF interpreting the way in which an issue was framed by the authors of individual reviews. Thirdly, as is common in framework synthesis, further synthesis was needed because certain facilitators and barriers arose at specific stages during implementation and this was not captured by the CFIR [31]. This is because the CFIR organises factors through the different constructs only. Therefore, using an iterative process, the review team identified the importance of different stages of the implementation process inductively from both the extracted data and their knowledge of implementation science; discussing, debating and reflecting on the issues identified. This involved regularly referring back to the extracted data and full text copies of the reviews [33]. As other implementation theories and frameworks were read, the ‘Knowledge to Action Framework’ was particularly relevant to the aspects of the extracted data that were not captured by the CFIR [34]. This additional framework highlighted that implementation involves phases of action and using this idea, the phases of implementation was developed inductively from the data extraction. The coding and synthesis were comparable to that used in qualitative research; similar techniques were used to ensure rigour including the use of an audit trail, double extraction, critical discussion amongst the review team and a sensitivity analysis which entailed comparing the results with some of the publications not included in the review [33].

## Results

### Selection of reviews

**Fig. 1:**
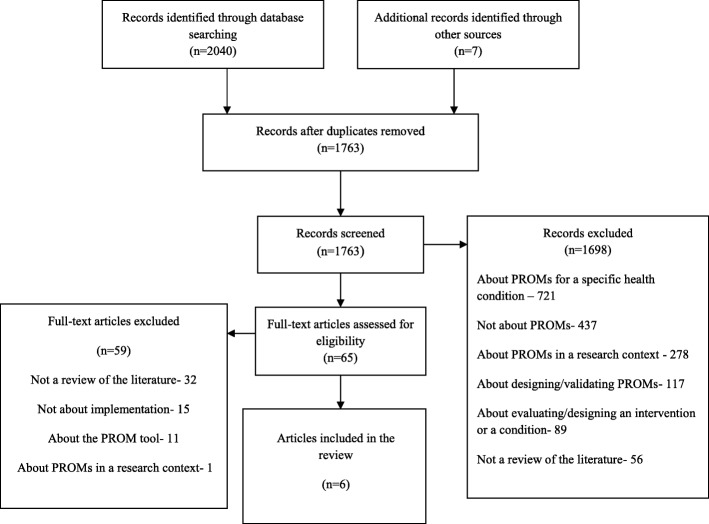
PRISMA Statement for the systematic review of reviews

Figure 1 presents the PRISMA flow diagram of study selection. Searches of the electronic databases yielded 2040 potentially relevant publications. Of these, 285 were duplicates and removed. Seven additional publications were identified through other search methods: four through contact with researchers and three through reference searching. After reviewing the titles/abstracts of the 1763 potential publications, 1698 were excluded. The three main reasons for exclusion were because a publication was a review of available PROMs to use for a specific health condition (*n* = 721), the review was not about PROMs (*n* = 437) or the review was about PROMs used in a research rather than routine practice context (*n* = 278).

After reviewing the full texts of 65 publications, 59 were excluded. Thirty-two publications were not formal reviews of the literature (for example they did not describe their search methods). Other reviews were excluded because they were not about implementation (*n* = 15); were focused on the measurement properties of PROMs (*n* = 11) or were about using PROMs in a research context (n- = 1). Of the 15 reviews excluded because they were not about implementation, 12 focused on the impact of PROMs, such as whether they improved clinical outcomes or patient satisfaction. The review team were initially uncertain about whether to include these, but ultimately excluded them because they were not about implementation in routine practice.

### Characteristics of the included reviews

Six reviews, published from 2012 to 2017 were included in the synthesis [22–24, 35–37]; a description of their characteristics is provided in Table 2. The reviews provided an international perspective, including studies from South Asia, the Middle East, Europe, Australasia and the Americas and review teams based in the UK, Ireland and Canada.

**Table 2:** Description of the reviews

First author and year	Setting	Aims	Type of review	Synthesis methods	Inclusion criteria for individual studies	Exclusion criteria for individual studies	Number of individual articles/reports included
Antunes, 2014 [22]	Palliative care	Identify barriers and facilitators to implementing PROMs in palliative care settings and generate recommendations to inform the process.	Systematic review	Narrative synthesis	(a) Primary studies published in English, Portuguese, Spanish, Italian, German and French. (b) Studies using a PROM alongside the clinical care of adult patients in palliative care settings. (c) Studies reporting barriers and/or facilitators of implementing PROMs.	(a) Published literature other than primary studies. (b) Studies reporting on the development and feasibility of specific PROMs. (c) Studies of PROMs not completed by the patient e.g. completed by a carer.	31
Bantug, 2016 [36]	Any healthcare setting	Identify information on the graphical display of PROMs data in routine practice.	Integrative review	Synthesis through generating themes	(a) Reported primary studies. (b)Addressed the communication of PROMs data to patients or clinicians. (c) Published between 1999 and 2014. (d) Published in either English or French.	No exclusion criteria specified.	9
Boyce, 2014 [23]	Any healthcare setting	Identify the barriers and facilitators for clinicians in using the information generated from PROMs.	Systematic review	Thematic synthesis	(a) Studies published in English. (b) Participants were clinicians. (c) Studies examined clinicians’ views of PROMs after receiving feedback. (d) Studies used a qualitative methodology.	No exclusion criteria specified.	16
Duncan, 2012 [35]	Care provided by Allied Health Professionals	Identify the barriers and facilitators to using PROMs in routine practice by Allied Health Professionals.	Systematic review	Narrative analysis	(a) Studies concerned with identifying facilitators/barriers in the routine use of PROMs by Allied Health Professionals in practice. (b) Studies published in English.	(a) If the topic in the studies was not of direct relevance. (b) If samples were not clearly defined. (c) If a sample was not wholly composed of Allied Health Professionals.	15
Greenhalgh, 2017 [24]	Any healthcare setting	Identify the processes through which, and circumstances in which, PROMs feedback improves patient care.	Realist synthesis	Realist synthesis	(a) Studies which provided a theoretical framework that describes how the process of feeding back individual PROMs intends to work. (b) Studies which provided a critique, review or discussion of the ideas underlying how individual PROMs feedback is intended to work. (c) Studies that provided stakeholder accounts or opinions of how individual PROMs feedback does/does not work. (d) Studies which outlined, discussed or reviewed potential unintended consequences of individual PROMs feedback.	(a) If studies focused on PROMs as a research tool. (b) If studies focused on evaluating or reviewing the psychometric properties of PROMs. (c) If studies provided advice or recommendations for which PROM to use in a research context.	36
Howell, 2015 [37]	Cancer care	Identify the PROMs used within routine cancer services, their impact and the factors influencing uptake.	Scoping review	Does not specify which method used	(a) Studies which reported on the routine use of PROMS. (b) The PROM was completed by the patient. (c) Included cancer patients or survivors. (d) Evaluated outcomes at the patient, clinical practice or care process or system level or barriers/enablers to using PROMs. (e) Studies published from 2003. (f) Studies published in English. (g) Could be primary quantitative or qualitative studies or systematic literature reviews.	No exclusion criteria specified.	30 individual studies and 4 systematic reviews.

Four types of review were included: systematic reviews [22, 23, 35], a realist synthesis [24], a scoping review [37] and an integrative review [36]. All six used a form of qualitative synthesis, for example narrative synthesis [22]. Three reviews focused on a specific area of healthcare: palliative care [22], cancer services [37] and services delivered by Allied Health Professionals [35]. The other three reviews focused on a particular aspect of the PROMS process: graphical display of data [36] and using the information generated from PROMs [23, 24]. These reviews included studies from a range of clinical settings. One review [35] considered PROMs as part of a wider focus on outcome measures. Reviews were included from a range of settings in order to get an overview of issues with different parts of the implementation process, occurring in different healthcare contexts.

The number of individual studies in each review ranged from 9 to 36 studies. Cumulatively, 118 individual studies were included within the reviews. There was little crossover between the reviews, with only 15 of the individual studies included in two or more of the reviews (13%). One review [37] also included four systematic reviews (none of which are included in this review because they did not meet the eligibility criteria). The interpretation of individual studies included in two or more reviews was broadly consistent.

### Risk of bias within the reviews

Whilst the ROBIS framework was used to assess risk of bias (see Table 3), the tool was not useful for comparing bias across the six reviews because some of the assessment topics were not applicable to scoping, realist or integrative reviews [24, 36, 37]. The systematic reviews which undertook all of the processes assessed by the ROBIS were scored as having a low risk of bias [22, 23, 35]. As none of the reviews had a high risk of bias, the ROBIS scores were not considered when synthesising the findings.

**Table 3:** Risk of Bias Assessment (ROBIS) of the reviews

	Author					
	Antunes, 2014 [22]	Bantug, 2015^b^ [36]	Boyce, 2014 [23]	Duncan, 2012 [35]	Greenhalgh, 2017^c^ [24]	Howell, 2015^d^ [37]
Domain 1: Concerns regarding specification of study eligibility criteria	Low	High- No description of the exclusion criteria	High- No description of the exclusion criteria	Low	Low	High- No description of the exclusion criteria
Domain 2: Concerns regarding methods used to identify and/or select studies	Unclear- No information on whether more than one researcher supported the search process	Low	Unclear- No information on whether more than one researcher supported the search process	Unclear- No information on whether more than one researcher supported the search process	High- Sought to identify studies which supported/challenged programme theories rather than identify all the available literature	High- No searching beyond electronic databases
Domain 3: Concerns regarding methods used to collect data and appraise studies	Low	High- No quality appraisal	Low	Low	High- Did not synthesis all relevant studies nor conduct quality appraisal because of it being a realist synthesis	High- Lack of information on which studies were included or description of the studies. No quality appraisal
Domain 4: Concerns regarding the synthesis and findings	Low	Low	Low	Low	High- As did not include all relevant studies there are issues with the synthesis	High- Concerns about the synthesis for example it was not clear which studies were included in the synthesis
Did the interpretation of findings address all of the concerns identified in Domains 1 to 4?	Probably yes	Probably no	Probably yes	Probably yes	Probably yes	Probably no
Was the relevance of identified studies to the review’s research question appropriately considered?	Yes	Yes	Yes	Yes	Yes	Probably yes
Did the reviewers avoid emphasizing results on the basis of their statistical significance?^a^	Yes	Yes	Yes	Yes	Yes	Probably no
Overall risk of bias in the review	Low	Unclear	Low	Low	Unclear	Unclear

‘Probably’- On the ROBIS there is the option to select ‘probably yes’, or ‘probably no’ in cases where the reviewer is not entirely sure. For example if it appeared that a review considered the relevance of the studies it included but the review did not include all the information on this to make the reviewer certain

^a^The ROBIS considers statistical significance but because the reviews are qualitative this question should be whether a review presented all its findings rather than cherry picking the results

^b^Please note that Bantug (2016) [36] was an integrative review so would not have undertaken some elements assessed by the ROBIS

^c^Please note that Greenhalgh (2017) [24] was a realist synthesis so would not have undertaken some elements assessed by the ROBIS such as including all relevant articles

^d^Please note that Howell (2015) [37] was a scoping review so would not have undertaken some elements assessed by the ROBIS such as quality appraisal

### Synthesis of results- barriers and facilitators to implementing PROMs

**Fig. 2:**
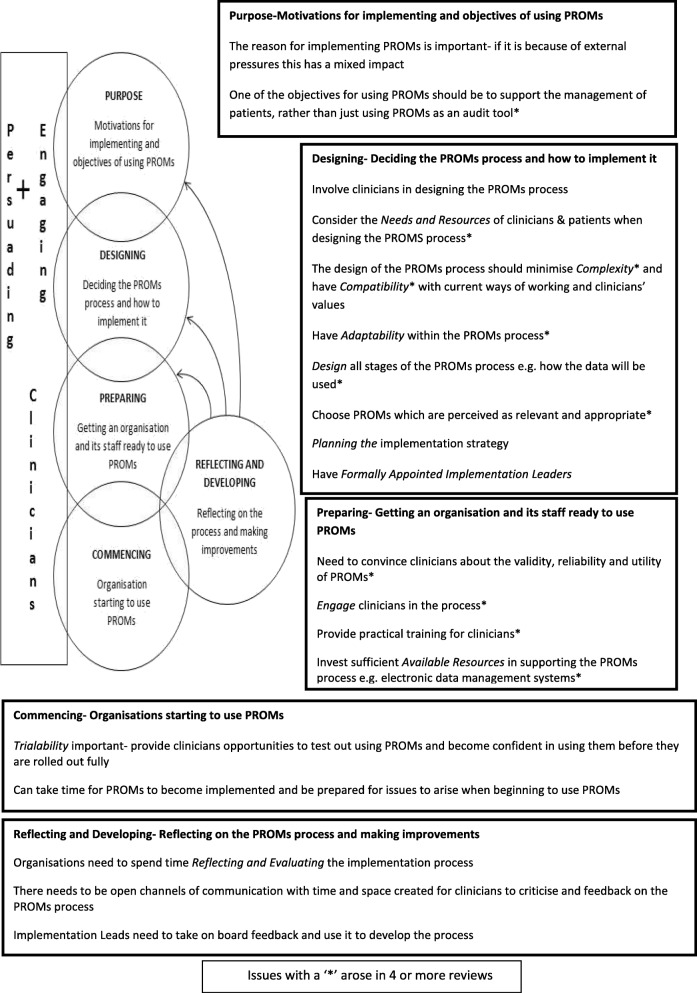
Facilitators by stage of PROMS implementation

The facilitators and barriers identified in the reviews are presented using the CFIR constructs (Additional file 3: Table S1). As explained in the methods section, the data was further categorised into five stages of implementation: Purpose, Designing, Preparing, Commencing, and Reflecting and Developing (Fig. 2). Some of the CFIR constructs were relevant to specific stages (displayed in italics e.g. *External Policies and Incentives*). Other CFIR constructs transcended several stages and have been developed to reflect this. Many factors were bidirectional, so they could be a facilitator or barrier depending on their execution. Given this, Fig. 2 focuses on facilitators only. In Fig. 2, facilitators identified in four or more reviews, are denoted with an ‘*’. As these facilitators have been identified in a number of reviews and thus diverse contexts, there is a greater chance that they may be relevant to other healthcare settings than issues identified in just one review.

#### Stage 1 defining ‘purpose’– How the motivations for, and objectives of, using PROMs impact on implementation (see Fig. 2)

There were different motivations for utilising PROMs and these impacted on implementation differently. Aligning PROMs with *External Policies* such as clinical practice guidance facilitated their use because it meant that clinicians perceived PROMs as part of their professional practice [37]. However, the use of what the CFIR terms *External Incentives* could be a barrier [24, 35]. For example, when the purpose of PROMs was to satisfy the demands of an external agency, there may be gaming of the data [24].

Implementation was facilitated when the objective was to use PROMs at an individual patient level to support patient-centred care. However, collating PROMs scores across a number of patients served as a barrier to implementation when the aim was to monitor clinical performance rather than provide useful information to clinicians on their patients [23, 24, 37]. The need for PROMs to be useful at an individual patient level appeared to be relevant across different healthcare settings.

#### Stage 2 ‘designing’- how the design of the PROMs process impacts on implementation (see Fig. 2)

##### Designing the PROMs process

The designing stage encompasses decisions about the choice of PROM tool and the processes for gathering, managing, interpreting and acting on the data generated from PROMs. Five of the reviews identified that the choice of PROM tool had a bidirectional impact on implementation. Choosing a PROM which clinicians perceived as valid, relevant and useful to their work facilitated implementation [22], as did selecting a PROM that clinicians perceived to be user-friendly, for both them and their patients [23, 24, 35]. However, *Costs* associated with using a PROM, such as prohibitive licence fees, could prevent an appropriate PROM being used [22]. Ensuring that patients received support to complete PROMs [24] and investing in technological solutions such as web-based apps facilitated implementation because it decreased the burden on clinicians and administrators [23]. Patient Viewpoint, an electronic system for supporting the completion and management of PROMs [38] was provided as a good practice example in one review [24]. Designing processes that enabled clinicians to utilise the PROMs data in their work [22–24, 36], such as reporting systems that produced easy to understand graphs of patients’ data [36], facilitated interpretation and thus use of the data.

A key facilitator when designing the PROMs process was ensuring *Adaptability,* both to the organisational context and to specific patients. This included having flexibility on if, when and how to administer a PROM to a patient [22–24, 35]. Designing a process which had *Compatibility* with clinicians’ values and organisational work flows facilitated implementation; for example, aligning data collection with appointment schedules [22–24, 35, 37]. If the process designed was perceived as having *Complexity,* this was a barrier [22–24, 35–37]. These factors, along with the implementation process generally was facilitated by involving clinicians in the designing stage [22].

All of the reviews identified that it was important to consider *Patients’ Needs and Resources* [22–24, 35–37] when designing the PROMs process. This entailed both the actual needs of patients, but also clinicians’ perceptions of their needs. Actual needs included choosing an appropriate PROM for patients’, and ensuring the process was flexible to their needs. Perceived needs included whether clinicians felt that patients would benefit or be disadvantaged by completing PROMs, such as their care regime being altered [22, 23]. Two reviews discussed consulting patients about which PROMs to use [24, 37]. However none of the reviews reflected on whether involving patients in designing the PROMs process facilitated implementation.

##### Planning the implementation process

*Planning* the implementation process [22, 36] and having *Formally Appointed Internal Implementation Leads* who manage the process in a sensitive and supportive manner facilitated implementation [22, 23].

#### Stage 3- ‘preparing’– Investing time and resources in preparing an organisation and clinicians to implement PROMs (see Fig. 2)

The reviews identified that investing sufficient time and resources to ensure an organisation’s *Readiness for Implementation* was a facilitator*.* All of the reviews discussed the bidirectional impact of clinicians’ *Knowledge and Belief*s on the implementation of PROMs]and that it was important for organisations to invest time and resources in *Engaging* and persuading clinicians on the value of using PROMs]. This included providing training] which conveyed the validity of PROMs as well as the benefits and justification for using them [22–24, 35–37]. Practical training needed to cover administering PROMs, analysing and interpreting the data, and managing issues arising from the PROMs.

Several of the reviews identified that organisations needed to invest sufficient *Available Resources* in systems to support the PROMs process. Examples include electronic databases, which can be used to record, manage and use the PROMs data; sufficient administrative support to process PROMs and having services available to address any clinical issues identified from PROMs scores [22–24, 35].

The reviews generally focused on organisations preparing clinicians and investing resources, taking the perspective that implementation was driven by an organisation and its leadership, and that it was clinicians who needed persuading to use PROMs. Arguably it could be the reverse, that a clinician wants PROMs to be implemented but the organisational culture is not receptive to change. Whilst two reviews considered the need for managers to be engaged and lead the implementation process [22, 23], the reviews did not give much consideration to the inner setting of organisations. That is, how the organisational culture and structural characteristics of organisations impacted on implementation. This differs to the CFIR, which has a number of constructs related to these issues. Notably, there was nothing in the reviews regarding preparing patients for the introduction of PROMs.

#### Stage 4- ‘commencing’- the issues that arise when starting to use PROMs (see Fig. 2)

The reviews identified a number of barriers that arose when *Executing* the implementation of PROMs. These were that it takes time and effort for PROMs to become a routine part of practice [22], the burden may fall on a small proportion of clinicians [23] and often problems arise when starting to use PROMs such as adapting it to individual patients [24]. For example, some patients may struggle to complete the PROMs. This relates back to the idea of having *Adaptability* when designing the PROMs process, so that clinicians have both flexibility and discretion in how they utilise the PROMs with specific patients. *Trialability*, which involves user-testing, in terms of clinicians piloting PROMs with a small number of patients, may facilitate the commencement of PROMs [23]. The reviews did not consider commencing in great detail, raising questions about how relevant these issues are across different healthcare contexts.

#### Stage 5- ‘reflecting and developing- reflecting on the PROMs process and making improvements (see Fig. 2)

Reflecting and Developing occurred when organisations gave their staff opportunities to provide constructive feedback, and then used the feedback to develop the PROMs process. However, as this facilitator was only raised in the reviews on palliative care and with allied health professionals [22, 35]; it raises questions about how relevant this stage is to other contexts.

## Discussion

### Summary of findings

This review identified a number of bidirectional factors arising at different stages which impact on the implementation of PROMs. Investing time and resources during the ‘designing’ and ‘preparing’ stages was important.. The designing stage involved organisations planning not just which PROMs to use and how to administer them, but also how the data would be managed and used for clinical purposes. The preparing stage involved getting an organisation and its staff ready to use PROMs. A key aspect of this stage was providing clinicians with training, including on the validity and value of PROMs. Organisations needed to invest in systems and resources to support the PROMs process such as electronic databases and administrative staff. Identifying individuals to lead the implementation and reflecting and developing the process based on feedback also facilitated implementation.

The stages of implementation were developed from the static constructs of the CFIR. Some constructs related to specific implementation stages, whereas others transcended several stages. Firstly, the constructs of the *Intervention Characteristics* domain formed the designing stage. Secondly, the constructs of *External Settings* varied; *Patients’ Needs and Resources* generally related to the designing stage, whereas *External Policy and Incentives* were part of the purpose stage. The constructs of the domains of *Inner Setting* and *Personal Characteristics* related to the preparing stage. Finally, the constructs related to the domain of *Process* transcended the stages.

Healthcare services are diverse and it is paramount to consider whether they incur different barriers and facilitators. Included reviews either focused on a specific clinical speciality or on a single part of the PROMs process, but within a range of clinical contexts. There were no contradictions between the reviews. However, there were some factors which were identified in some of the reviews but not others. This may be genuine differences or be due to the reviews having different remits. Therefore there is a need for further research comparing the whole implementation pathway across different healthcare contexts [35].

Whilst one review gave examples of implementation [24], this was limited to parts of the process rather than examples of a whole implementation pathway. Consequently, there is a gap in knowledge about how the individual factors interrelate and influence implementation. None of the reviews considered causality, so the actual impact of any of the identified issues on the implementation of PROMs is unknown. For example, is the provision of training to clinicians associated with the proportion of patients who complete PROMs?

### Comparison with other literature

The findings of this review were compared with a range of existing literature including other studies focused on implementing PROMs, especially publications not included in this review; published guidance on implementing PROM and literature on implementing other types of clinical performance measures.

The findings of this review are generally consistent with other literature which explores the implementation of PROMs. However, this review places greater emphasis on the whole implementation pathway, whereas much of the previous literature primarily focuses on the designing stage.

This review found that the purpose for using PROMs influenced implementation, in that it was important for organisations to find ways of making PROMs useful for clinicians. This is consistent with the wider literature on changing clinical practice, which notes that clinicians need to understand how they would benefit from any change in practice [39].

Similar to other literature, this review highlighted the importance of investing time in designing the PROMs process] and tailoring it to the specific context by considering the needs and opinions of patients and clinicians [40]. This review identified that it was useful to involve clinicians in designing the PROMs process and this has been identified in other studies [41]. From both this review and wider literature it is evident that organisations not only need to design how to administer PROMs, but also consider how clinicians should interpret and act on the data generated].

Whilst other publications have identified that it is important to train clinicians on the practical elements of using PROMs [15, 19, 20, 42], the findings of this review place greater emphasis on the need to engage and persuade clinicians to use PROMs. This review did not consider whether the engagement work needed to be ongoing until culture change occurred, whereas a recent study identified that engagement work was an ongoing process [43].

As other studies have found, there is a need for organisations to invest in electronic systems [44]. However, this may not always be feasible, for example due to budget constraints or a lack of prioritisation by the organisation. This raises questions about how implementation is impacted if an organisation cannot adopt all of the facilitators or address the barriers. For example one review suggested that if an organisation cannot choose which PROM to use, it could compensate for this by undertaking greater engagement work with clinicians]. This idea needs further exploration to understand if and when compensation strategies can be effectively used.

The CFIR includes a number of inner setting constructs which focus on the structural characteristics of an organisation [10], such as the impact of the size of an organisation. However, there was little focus in the reviews on these inner setting constructs. The reviews did not consider how to sustain the use of PROMs after the initial intervention activities [45]. This is in contrast to a recent study which raised questions about the sustainability of PROMs, for example due to a lack of investment in infrastructure [43]. Further research is needed to explore whether the use of PROMs is sustained in organisations after implementation strategies cease.

Whilst this review identified that patients’ needs should be considered when implementing PROMs, there was little in the reviews about involving patients with designing the PROMs process. This contrasts with other literature that emphasises the need to involve patients in designing PROMs [46], and arguably the whole implementation process.

A sensitivity analysis revealed that this reviews’ findings were generally consistent with the findings from publications that had been excluded from this review because they were not considered formal reviews of the literature. However the other reviews did place a greater emphasis on the advantages of using electronic methods to administer PROMs, but with the caveat of organisations needing sufficient technological support [47]. An additional finding from these excluded publications was the identification of issues which influence implementation in resource-limited countries [4]. This expands the debate as highlights the potential role of wider contextual factors such as country of delivery.

Alongside reviews, guidance on implementing PROMs has been published. One example is generic to healthcare services [48], whereas another is specific to palliative care [49]. The generic guidance focuses on designing the PROMs process [48], whereas the palliative care guidance is more aligned with the findings of this review. This is because it takes into account the wider implementation pathway such as engaging clinicians [49]. This difference could be because as with this review, the palliative care guidance has been developed recently and therefore incorporates current knowledge on implementation.

Research on implementing other clinical performance measures, such as audits, have had similar findings to this review [50, 51]. Key facilitators were using performance measures which relate to the quality of care rather than productivity, considering patients’ needs, having flexibility in the process and utilising electronic systems [50, 51]. However, unlike this review, training was not always found to impact on implementation. [50]. The literature on implementing clinical performance measures has explored the impact of the structural characteristics of organisations. Whilst the evidence is inconclusive, it highlights that structural characteristics need to be considered in relation to implementing PROMs [50].

### Strengths and limitations

This systematic review of reviews appears to be the first review to synthesise knowledge across different clinical specialities and the whole implementation pathway, identifying the cross-cutting issues with PROMS implementation. Using the CFIR provides the review with a theoretical underpinning, which is missing in much of the literature on PROMS implementation. The CFIR was generally a ‘good fit’ with the findings of the review [52], helping to make sense of the data and highlighting aspects of implementation not explicitly identified within the individual reviews. The review develops the findings beyond the static constructs of the CIFR through framing them within stages of implementation (Fig. 2). Taking this approach helps to communicate the dynamic nature of implementation, and could be used by people implementing PROMs. Finally, a general criticism of systematic reviews of reviews is that several reviews may include the same individual studies. However, in this case, only 13% of the individual studies were included by two or more of the reviews. The review findings include 113 unique individual studies, enabling synthesis of implementation issues across different settings.

This review had six limitations. Firstly, there may be useful individual studies that have not been included in a published review and thus are not considered in this review.. This limitation was addressed by considering whether the findings were similar to those raised within the wider literature, including individual studies. Secondly, there were limitations in the search methods used. English language restrictions were applied due to a lack of resources to translate articles. This excluded 3.3% of the titles identified in the MEDLINE search. Given this fairly small number it is unlikely that key reviews were missed. The non-electronic database search methods were UK focused; this was because the review formed part of a study based on the UK context. Thirdly, 32 publications were excluded because they did not include any information on the review methods used, for example not specifying their search strategy. This is a common exclusion criteria for systematic reviews of reviews] and was addressed by performing a sensitivity analysis comparing this reviews findings’ with those of the excluded reviews (discussed above). Fourthly, the ROBIS lacked relevance to reviews not categorised as standard systematic reviews by their authors. This indicates the need for further methodological work on how to appraise different types of reviews. Fifthly, the synthesis of findings utilised a facilitators and barriers framework, which could mean other factors were excluded. However, to minimise this risk the search terms did not include ‘facilitators and barriers’ and issues were extracted irrespective of the specific terminology used. Finally, as the unit of analysis was the reviews themselves rather than individual primary studies, this review is reliant on how comprehensively the individual reviews extracted information on implementation from their primary studies. This has been addressed by comparing the findings with other literature.

### Implications

As many of the facilitators and barriers were identified in several of the reviews across different contexts, the findings may be relevant to other organisations wanting to implement PROMs. That is, organisations need to pay attention to the early stages of implementation of PROMs in terms of designing the PROMs process and preparing both clinicians and organisations. Some facilitators and barriers were only identified in one review and it is difficult to know if this is because they only occur in that specific context. This is salient because although the CFIR contains a number of constructs related to inner settings, such as the structural characteristics of an organisation, the reviews did not focus on these.. Future clinical practice and research needs to consider the impact of context on implementation, particularly considering the impact of clinical specialities, structural characteristics of organisations and types of healthcare services e.g. differences between community and inpatient care or publicly verses privately funded organisations. For example, if a smaller organisation does not have electronic data collection systems, do they have to compensate for this during the implementation process? The second issue in relation to context is that there were no reviews focusing on social care or third sector providers; these organisations will need to consider the extent that these findings are applicable to their organisational contexts. A key facilitator was the need for PROMs to be useful for clinicians, but there will be cases where PROMs are being used for other purposes such as performance management and further research is needed on how to persuade clinicians of the value of PROMs, to both themselves and their patients in this scenario. Increasingly patients, clinicians and other stakeholders are involved in co-producing healthcare interventions and there is a need for research on how co-production could facilitate the implementation of PROMs [41]. Both training and electronic systems are issues which need further exploration. Whilst this review and other literature have emphasised their importance in the implementation process, there is a need to draw upon exemplars to identify which specific features of training programmes and electronic systems facilitate the implementation of PROMs. This review focused on the initial implementation of PROMs, however further research is needed on sustaining their use, for example whether there is a need for ongoing engagement activities or how changes in organisational culture impact on PROMs becoming part of routine practice. Finally, this review has considered the different issues as independent components and there is a need for further research on how they may interact, whether some are more influential than others and if any have a causal impact on the implementation of PROMs.

## Conclusion

In conclusion, a range of factors have a bidirectional impact on the implementation of PROMs at different stages of the implementation pathway. Two crucial stages are designing the PROMs process and preparing an organisation for implementation, especially training clinicians. Both require time and resources. As the findings were generally consistent between the included reviews and with the wider literature, they are likely to be relevant for organisations implementing PROMs.

## Additional files

Additional file 1: PRISMA checklist. (DOC 58 kb)

Additional file 2: MEDLINE search terms. (DOCX 13 kb)

Additional file 3: **Table S1.** The facilitators and barriers to implementing PROMs [22–24, 35–37]. (DOCX 47 kb)

## Abbreviations

CFIR: Consolidated Framework for Implementation Research
PRISMA: Preferred Reporting Items for Systematic Reviews and Meta-Analyses
PROMIS: Patient-Reported Outcomes Measurement Information System
PROMs: Patient Reported Outcome Measures
PROs: Patient Reported Outcomes
PROSPERO: International prospective register of systematic reviews
ROBIS: Risk of Bias in Systematic Reviews
UK: United Kingdom
USA: United States of America
