# Supplementary material for: The facilitators and barriers to implementing patient reported outcome measures in organisations delivering health related services: a systematic review of reviews
Source: J Patient Rep Outcomes. 2018 Oct 3;2:46. doi: 10.1186/s41687-018-0072-3 (PMC6170512; doi:10.1186/s41687-018-0072-3)
Supplement: Supplementary file 2 — MEDLINE search terms. (DOCX 13 kb) [file 41687_2018_72_MOESM2_ESM.docx]

| 1 | (prom or proms or patient reported outcom* or pros or pro or prem or prems or patient reported experience measure* or epros or epro or outcome measure*).ti. |
| --- | --- |
| 2 | clinical adj (setting* or practice*).tw |
| 3 | ((routine adj2 collect* or outcome*)).tw. |
| 4 | (healthcare or social care or health care).tw. |
| 5 | Charit*.tw |
| 6 | (Voluntary sector or voluntary organi*).tw |
| 7 | Third sector.tw |
| 8 | (Grassroot* organi* or grassroot* project*).tw |
| 9 | (Community organi* or community project*).tw |
| 10 | Admin*.tw |
| 11 | Implement*tw |
| 12 | 2,3,4,5,6,7,8,9,10 or 11 |
| 13 | Meta anlysis.pt or meta anlysis.af |
| 14 | Review.pt |
| 15 | search:.tw. |
| 16 | 13, 14 or 15 |
| 17 | 1 and 12 and 16 |
| 18 | English.lg |
| 19 | 17 and 18 |

**Additional file 2- Search terms used on MEDLINE**
