# Supplementary material for: The facilitators and barriers to implementing patient reported outcome measures in organisations delivering health related services: a systematic review of reviews
Source: J Patient Rep Outcomes. 2018 Oct 3;2:46. doi: 10.1186/s41687-018-0072-3 (PMC6170512; doi:10.1186/s41687-018-0072-3)
Supplement: Supplementary file 3 — Table S1. The facilitators and barriers to implementing PROMs [22–24, 35–37]. (DOCX 47 kb) [file 41687_2018_72_MOESM3_ESM.docx]

| **Domain 1- Intervention Characteristics- Issues related specifically to the intervention such as its design or cost**  **Table S1 - The facilitators and barriers to implementing PROMs** | | | | | | | | | | | | | | | | | | | |
| --- | --- | --- | --- | --- | --- | --- | --- | --- | --- | --- | --- | --- | --- | --- | --- | --- | --- | --- | --- |
| **CFIR Construct** | **CFIR Subconstruct** | **Antunes (2014) [22]** | | **Bantug (2016) [36]** | | **Boyce (2014) [23]** | | **Duncan (2012) [35]** | | | **Greenhalgh (2017) [24]** | | | | **Howell**  **(2015) [37]** | | **Examples** | | **Facilitator/**  **Barrier/**  **Bidirectional** |
| **Intervention source**- *Whether the PROM and PROMs process was internally or externally developed* |  | X | |  | |  | |  | | |  | | | | X | | Involving clinicians and patients in the development of the PROM process so that it’s not perceived as externally imposed | | Facilitator |
| **Evidence strength and quality-** *Stakeholders belief in the validity of PROMs* |  | X | |  | | X | | X | | | X | | | | X | | Knowledge about PROMs especially views on their reliability and validity | | Bidirectional |
| **Relative advantage-** *Perception that implementing PROMs is better than alternative solutions* |  | Nothing in the reviews about this concept. | | | | | | | | | | | | | | |  | |  |
| **Adaptability-** *The extent the PROMs process can be adapted to meet local needs* |  | X | |  | | X | | X | | | X | | | |  | | An organisation being able to choose which PROM to use  Flexibility to adapt the PROMs process to the particular setting and individual patient | | Facilitator  Facilitator |
| **Trialability-** *Ability to test using PROMs before implementing fully* |  |  | |  | | X | |  | | |  | | | |  | | Clinicians being able to test out and become experienced in using PROMs before they are introduced fully | | Facilitator |
| **Complexity-** *Perceived difficulty of implementing and using PROMs* |  | X | | X | | X | | X | | | X | | | | X | | Nature of a specific PROM e.g. its complexity  The difficulty or ease of administrating PROMs  The difficulty or ease of interpreting the scores from PROMs  The complexity of communicating the results of PROMs because they are multi-dimensional and vary between PROMs | | Bidirectional  Bidirectional  Bidirectional  Barrier |
| **CFIR Construct** | **CFIR Subconstruct** | **Antunes (2014) [22]** | | **Bantug (2016) [36]** | | **Boyce (**  **2014) [23]** | | **Duncan (2012) [35]** | | | **Greenhalgh (2017) [24]** | | | | **Howell**  **(2015) [37]** | | **Examples** | | **Facilitator/**  **Barrier/**  **Bidirectional** |
| **Design-** *Perceptions about the quality of the design of PROMs and the PROMs process* |  | X | | X | | X | | X | | | X | | | | X | | **The PROMs**  Using a PROM which asks about relevant issues  Patients may prefer individualised PROMs  Issues with the wording of PROMs e.g. double questions or unfamiliar concepts  PROMs not perceived as user friendly e.g. not prioritising issues for patients  **Process of administering PROMs**  Clinicians prefer to use PROMs once they have built up rapport with a patient  Patients having support to complete PROMs  Investing in technological solutions for collecting PROMs  If the process for administering a PROM is script like, it can detrimentally impact on rapport  Home monitoring such as electronic PROMs can be intrusive and restrictive for patients.  Using electronic systems to flag up scores  Presenting PROMs data in appropriate ways e.g. the simplicity of the graphs | | Facilitator  Facilitator  Barrier  Barrier  Facilitator  Facilitator  Facilitator  Barrier  Barrier  Facilitator  Bidirectional |
| **CFIR Construct** | **CFIR Subconstruct** | **Antunes (2014) [22]** | | **Bantug (2016) [36]** | | **Boyce**  **(2014) [23]** | | **Duncan (2012) [35]** | | | **Greenhalgh (2017) [24]** | | | | **Howell**  **(2015) [37]** | | **Examples** | | **Facilitator/**  **Barrier/**  **Bidirectional** |
|  |  | X | | X | | X | | X | | | X | | | | X | | **(Continued from design)**  **Managing the data from PROMs**  Using electronic systems to flag up scores  Presenting PROMs data in appropriate ways e.g. the simplicity of the graphs | | Facilitator  Bidirectional |
| **Cost-** *Costs of implementing PROMs* |  | X | |  | | X | | X | | | X | | | |  | | Investment in computer systems and information management systems to support the PROMs process  Licence costs of PROMs  Opportunity cost of using PROMs- can be perceived as using consultation time which could be spent on other aspects of a patient’s care | | Facilitator  Barrier  Barrier |
| **Domain 3- External settings- Impact of elements external to the organisation** | | | | | | | | | | | | | | | | | | |  |
| **Patient needs and resources-** *Impact of patient needs on implementing PROMs* |  | X | | X | | X | | X | | | X | | | | X | | **Patients’ needs**  Selecting PROMS based on the needs of patients  Having flexibility within the PROMs process to take into account individual needs  **Patients’ knowledge**  Clinicians more willing to use PROMs if they perceive that patients’ understand them  Implementation of electronic PROMs impacted by patients’ technological ability  Patients concerned about making a bad clinical decision based on them misinterpreting PROMs data  Patients can struggle with interpreting PROMs data | | Facilitator  Facilitator  Facilitator  Bidirectional  Barrier  Barrier |
| **CFIR Construct** | **CFIR Subconstruct** | **Antunes (2014) [22]** | | **Bantug (2016) [36]** | | **Boyce**  **(2014) [23]** | | **Duncan (2012) [35]** | | | **Greenhalgh (2017) [24]** | | | | **Howell**  **(2015) [37]** | | **Examples** | | **Facilitator/**  **Barrier/**  **Bidirectional** |
|  |  | X | | X | | X | | X | | | X | | | | X | | **(Continued from patient needs and resources)**  Clinicians concerned about the ability of patients to complete the PROMs e.g. literacy issues  **Impact on patients**  Clinicians are more likely to use PROMs if they perceive them as being beneficial for patients  Clinicians concerned that the results may impact detrimentally on patient care  Clinicians concerned that specific questions on PROMs may cause distress to patients  Clinicians feel administering PROMs can have a detrimental impact on their relationship with a patient  Clinicians concerned that patients can become disheartened if their PROMs’ scores are not improving | | Barrier  Facilitator  Barrier  Barrier  Barrier  Barrier |
| **Cosmopolitanism-** *Degree an organisation is networked with other organisations* |  | Nothing in the reviews about this concept | | | | | | | | | | | | | | |  | |  |
| **Peer pressure-** *Influence of competition with other organisations* |  | Nothing in the reviews about this concept | | | | | | | | | | | | | | |  | |  |
| **CFIR Construct** | **CFIR Subconstruct** | **Antunes (2014) [22]** | | **Bantug (2016) [36]** | | **Boyce**  **(2014) [23]** | | **Duncan (2012) [35]** | | | **Greenhalgh (2017) [24]** | | | | **Howell**  **(2015) [37]** | | **Examples** | | **Facilitator/**  **Barrier/**  **Bidirectional** |
| **External policy and incentives-** *Influence of wider policy and funding on implementing PROMs* |  |  | |  | |  | | X | | | X | | | | X | | Aligning PROMs with clinical practice guidelines  Incentivising PROMs e.g. with funding can have mixed results on implementation  External agencies e.g. a funder imposing PROMs on an organisation | | Facilitator  Bidirectional  Barrier |
| **Domain 3- Inner settings- Impact of the organisations' characteristics** | | | | | | | | | | | | | | | | | | | |
| **Structural characteristics-** *The nature of the organisation* |  | Nothing in the reviews about this concept | | | | | | | | | | | | | | |  | |  |
| **Networks & communications-** *The nature of networks and channels communication within an organisation* |  | Nothing in the reviews about this concept | | | | | | | | | | | | | | |  | |  |
| **Culture-** *The culture within an organisation* |  |  | |  | |  | | X | | |  | | | |  | | Having a positive team culture and the resulting normative social pressure will help engage clinicians | | Facilitator |
| **Implementation climate-** *The absorptive capacity for change and shared receptivity within an organisation* |  | Nothing in the reviews about this concept specifically as the related facilitators and barriers are included in the subconstructs below. | | | | | | | | | | | | | | |  | |  |
| **Implementation climate** | **Tension for change-** *Extent that people perceive the need for change* |  |  | | X | | X | | | X | | |  | | | | Fear amongst clinicians that the results of PROMs will impact detrimentally on practice and patient care  Clinicians can perceive that the introduction of PROMs is suggesting that their communication methods are not sufficient | | Barrier  Barrier |
| **CFIR Construct** | **CFIR Subconstruct** | **Antunes**  **(2014) [22]** | | **Bantug**  **(2016) [36]** | | **Boyce**  **(2014) [23]** | | **Duncan**  **(2012) [35]** | | | **Greenhalgh (2017) [24]** | | | | **Howell**  **(2015) [37]** | | **Examples** | | **Facilitator/**  **Barrier/**  **Bidirectional** |
| **Implementation climate** | **Compatibility-** *How much the intervention fits with the individuals’ own values and existing systems* | X | |  | | X | | X | | | X | | | | X | | If PROMs can be incorporated naturally into the consultation  PROMs more positively viewed if their purpose is as a care management tool and perceived more negatively when they are being used as a performance management tool  Use of PROMs impacted by whether a clinician perceives it within their remit to deal with any issues that arise from the PROM  Concern that PROMs add to clinician’s workload and they do not have sufficient time to administer them and manage the issues they raise  Perception that PROMs can damage communication, narrow the focus of a consultation and be intrusive, impacting on rapport  Concerns about how to manage issues that arise between appointments if patients complete PROMs outside of consultations | | Facilitator  Bidirectional  Bidirectional  Barrier  Barrier  Barrier |
| **Implementation climate** | **Relative priority-** *Perception of the importance of implementation within the organisation* |  | |  | | X | |  | | | X | | | |  | | The support provided by an organisation to use PROMs can impact on implementation  Clinicians have to appreciate the patient’s perspective rather than purely the physiological perspective e.g. blood test results | | Bidirectional  Bidirectional |
| **CFIR Construct** | **CFIR Subconstruct** | **Antunes**  **(2014) [22]** | | **Bantug**  **(2016) [36]** | | **Boyce**  **(2014) [23]** | | **Duncan**  **(2012) [35]** | | | **Greenhalgh (2017) [24]** | | | | **Howell**  **(2015) [37]** | | **Examples** | | **Facilitator/**  **Barrier/**  **Bidirectional** |
| **Implementation climate** | **Organisations incentives and rewards-** *Incentives within the organisation to use PROMs* |  | |  | |  | | X | | | X | | | |  | | Incentives do not necessarily facilitate the use of PROMs  Issue of PROMs being used as a performance management tool with clinicians | | Barrier  Barrier |
| **Implementation climate** | **Goals and feedback-** *The extent the goals of PROMs are communicated* |  | |  | | X | |  | | |  | | | |  | | It needs to be clear what the goals of using PROMs are and that they are not contradictory | | Facilitator |
| **Implementation climate** | **Learning climate-** *The ethos within the organisation to develop their skills and try new approaches* | X | |  | |  | |  | | |  | | | |  | | Clinicians need to feel they can influence the PROMs process and that their opinions on PROMs are valued | | Facilitator |
| **Readiness for implementation- Tangible and immediate indicators of an organisation committed to its decision to implement** |  | Nothing in the reviews about this concept specifically as the related facilitators and barriers are included in the subconstucts below. | | | | | | | | | | | | | | |  | |  |
| **CFIR Construct** | **CFIR Subconstruct** | **Antunes (2014) [22]** | | **Bantug (2016) [36]** | | **Boyce (2014) [23]** | | **Duncan(2012) [35]** | | | **Greenhalgh (2017) [24]** | | | | **Howell**  **(2015) [37]** | | **Examples** | | **Facilitator/**  **Barrier/**  **Bidirectional** |
| **Readiness for implementation** | **Leadership engagement-**C*ommitment and accountability of managers to implementation* | X | |  | | X | |  | | |  | | | |  | | Having sensitive and supportive leadership to motive people to use PROMs  Managers and leaders are involved in the process and are appreciative and acknowledge the additional work PROMs bring | | Facilitator  Facilitator |
| **Readiness for implementation** | **Available resources-** *T*h*e level of resources dedicated for implementation and ongoing operations* | X | |  | | X | | X | | | X | | | |  | | Providing sufficient administrative support  Having sufficient systems in place so that clinicians can refer onto other services/offer additional support if issues are raised from the PROMs  Sufficient investment in resources such as technology to help with the collection and analysis of PROMs  Whether there is sufficient statistical support to appropriately analyse and interpret the PROMs data | | Facilitator  Facilitator  Facilitator  Bidirectional |
| **Readiness for implementation** | **Access to knowledge and information-** *Ease of access to information and knowledge about PROMs and how to incorporate it into one’s work* | X | | X | | X | | X | | | X | | | | X | | Delivering good training to clinicians both in terms of the reasons for using PROM and how to use them e.g. practical issues  A lack of clear guidance about the process can be a barrier e.g. which patients should be completing a PROM | | Bidirectional  Barrier |
| **CFIR Construct** | **CFIR Subconstruct** | **Antunes**  **(2014) [22]** | | **Bantug**  **(2016) [36]** | | **Boyce**  **(2014) [23]** | | **Duncan**  **(2012) [35]** | | | **Greenhalgh**  **(2017) [24]** | | | | **Howell**  **(2015) [37]** | | **Examples** | | **Facilitator/**  **Barrier/**  **Bidirectional** |
| **Domain 4- Characteristics of individuals- How individuals impact on the process** | | | | | | | | | | | | | | | | | | | |
| **Knowledge and beliefs about the intervention-** *Individuals’ attitudes and value placed on the intervention* |  | X | | X | | X | | X | | | X | | | X | | | | Clinicians more supportive of PROMs if they perceive that PROMs have clinical utility e.g. enhancing communication  Managers employing clinicians who already use or are supportive of PROMs  Whether clinicians perceive that PROMs produce a genuine reflection of care e.g. their validity  Clinicians are the driving force in implementing PROMs and their values and beliefs on PROMs impact on their implementation  PROMs may not be valued by clinicians, who can perceive them as a tick box exercise  Clinicians may perceive PROMs as detrimentally impacting on their practice e.g. communication with a patient or narrowing the focus of the consultation  Clinicians may be resistant to having feedback from the PROMs scores and adapting their practice | Facilitator  Facilitator  Bidirectional  Bidirectional  Barrier  Barrier  Barrier |
| **Self-efficacy-** *Individuals belief in their own ability to execute courses of action to achieve implementation of PROMs* |  | X | |  | |  | |  | | |  | | |  | | | | Whether clinicians feel comfortable with using PROMs | Bidirectional |
| **CFIR Construct** | **CFIR Subconstruct** | **Antunes**  **(2014) [22]** | | **Bantug**  **(2016) [36]** | | **Boyce**  **(2014) [23]** | | **Duncan**  **(2012) [35]** | | | **Greenhalgh**  **(2017) [24]** | | | **Howell**  **(2015) [37]** | | | | **Examples** | **Facilitator/**  **Barrier/**  **Bidirectional** |
| **Individuals stage of change-** *Where an individual is at in terms of accepting the intervention* |  | X | |  | |  | | X | | |  | | |  | | | | Clinicians feeling they have ownership over the process  Clinicians feeling they have personal responsibility for using PROMs  Each individual clinician has their own emotional and cognitive processes which impacts on them using PROMs  Clinicians can have a fear of change | Facilitator  Facilitator  Bidirectional  Barrier |
| **Individual identification with an organisation-** *People’s relationship with an organisation and their commitment to that organisation* |  | Nothing in the reviews about this concept | | | | | | | | | | | | | | | |  |  |
| **Other personal attributes-** *Other aspects about clinicians which can impact on implementation* |  |  | |  | |  | | | X | | |  | |  | | | | Use of PROMs higher amongst clinicians with Masters qualifications or with clinical specialities. | Facilitator |
| **CFIR Construct** | **CFIR Subconstruct** | **Antunes (2014) [22]** | | **Bantug (2016) [36]** | | **Boyce (2014) [23]** | | | **Duncan (2012) [35]** | | | **Greenhalgh (2017) [24]** | | | | **Howell**  **(2015) [37]** | | **Examples** | **Facilitator/**  **Barrier/**  **Bidirectional** |
| **Domain 5- Process- The process of implementing PROMs** | | | | | | | | | | | | | | | | | | |  |
| **Planning**- *Extent that the implementation process is planned* |  | X | | X | |  | |  | | |  | | | |  | | | Good planning and clear boundaries from the start about expectations regarding PROMs  Consider the implementation of processes for managing and using the PROMs data | Facilitator  Facilitator |
| **Engaging-** *Engaging staff in both the implementation and intervention* |  | X | | X | | X | | X | | | X | | | | X | | | Having well-designed training  If PROMs are imposed then there is a greater need to be proactive in engaging clinicians  Engaging clinicians as a way of convincing them of the value of PROMs | Facilitator  Facilitator  Facilitator |
| **Engaging** | **Opinion leaders-** *Individuals within an organisation that have influence on their colleagues* | X | |  | |  | |  | | |  | | | |  | | | Having sensitive leadership to motivate individuals and reassure them about the value of PROMs |  |
| **Engaging** | **Formally appointed internal implementation leaders-** *Individuals who have responsibility for implementing* | X | |  | |  | |  | | |  | | | |  | | | There is a need to have someone in charge of the process of implementing PROMs  It can be useful for the Implementation Lead to use a cascade management style | Facilitator  Facilitator |
| **CFIR Construct** | **CFIR Subconstruct** | **Antunes (2014) [22]** | | **Bantug (2016) [36]** | | **Boyce (2014) [23]** | | **Duncan (2012) [35]** | | | **Greenhalgh (2017) [24]** | | | | **Howell**  **(2015) [37]** | | | **Examples** | **Facilitator/**  **Barrier/**  **Bidirectional** |
| **Engaging** | **Champions-** *People who drive implementation* |  | |  | |  | | X | | |  | | | |  | | | Having the support and co-operation of colleagues and managers is important | Facilitator |
| **Engaging** | **External change agents-** *People outside of the organisation influence the implementation of PROMs* | Nothing in the reviews about this concept | | | | | | | | | | | | | | | |  |  |
| **Executing-** C*arrying out the implementation of PROMs according to the implementation plan* |  | X | |  | | X | |  | | | X | | | |  | | | Integrating a new routine into daily clinical practice takes time and effort  There can be issues if it is perceived that the burden of collecting PROMs is falling on one or two members of the team    Problems can arise when PROMs start to be used e.g. gaming of the data | Barrier  Barrier  Barrier |
| **CFIR Construct** | **CFIR Subconstruct** | **Antunes (2014) [22]** | | **Bantug (2016) [36]** | | **Boyce (2014) [23]** | | **Duncan (2012) [35]** | | | **Greenhalgh (2017) [24]** | | | | **Howell**  **(2015) [37]** | | | **Examples** | **Facilitator/**  **Barrier/**  **Bidirectional** |
| **Reflecting and evaluating-** *Evaluation of the implementation* |  | X | |  | |  | | X | | |  | | | |  | | | Review the implementation process and make changes as needed to the PROMs process  During the implementation process have open channels of communication so that everyone can provide constructive criticism  Leaders need to take account of the constructive criticism and address the issues raised  Develop an evaluation plan to assess the impact of collecting PROMs  Clinicians can be blamed if PROMs are not utilised but there may be multi-level determinants that can be contributing and if these are not addressed then the implementation of PROMs will continue to fail | Facilitator  Facilitator  Facilitator  Facilitator  Barrier |
